# Knowledge of UK Residents About Importing Puppies from EU Countries †

**DOI:** 10.3390/ani15152193

**Published:** 2025-07-25

**Authors:** Zoe Belshaw, Rowena M. A. Packer

**Affiliations:** 1 EviVet Evidence-Based Veterinary Consultancy, Nottingham NG2 5HY, UK; z.belshaw.97@cantab.net; 2 Department of Clinical Science and Services, The Royal Veterinary College, Hawkshead Lane, North Mymms, Hatfield, Herts AL9 7TA, UK

**Keywords:** dog, puppy, welfare, acquisition, purchasing, import, foreign disease, zoonoses, veterinary, legal

## Abstract

The number of puppies imported to the United Kingdom (UK) from the European Union (EU) is increasing, as are concerns about the legality, welfare and potential disease risks associated with their trade. This study aimed to use an online survey to collect questionnaire data from UK residents about their awareness and knowledge of EU Pet Passports, illegal puppy importation, and dog-borne exotic diseases and the risks they might pose to UK-resident dogs and people. Valid responses were collected from *n* = 7184 UK residents, mainly current dog owners. We identified substantial knowledge gaps relating to EU Pet Passports, how puppies might be moved across Europe, and the legal age of importation. Awareness of exotic diseases was poor; knowledge about the risks they posed was even poorer, particularly in relation to dog–human transmission. With some respondents not even being confident that they had heard of rabies, there is substantial work to do to ensure that the UK canine and human populations remain safe in the face of significant welfare and exotic disease threats. These knowledge deficits may facilitate the burgeoning illegal trade in puppies across the EU. Legal change may be required to prevent consumers from unintentionally supporting poor welfare sources of puppies.

## Simple Summary

There is growing concern about the number of puppies being imported to the United Kingdom (UK) from European countries such as Romania; up to 10% of puppies purchased in the UK during the COVID-19 pandemic are thought to have originated in the European Union (EU). The imported puppy trade is associated with substantial illegality, poor traceability and potential for substantial welfare detriment to the puppies involved. Little previous research has been conducted into UK residents’ knowledge of the logistics, legalities and disease risks associated with puppies being imported to the UK from the EU. This research aimed to fill that knowledge gap using a UK-wide survey. Valid responses were analyzed from 7184 respondents, most of whom were current dog owners. We identified significant knowledge gaps relating to what is conferred by an EU Pet Passport, how puppies are transported from Romania to the UK, and about the existence and transmission risks associated with a range of exotic diseases. These knowledge gaps may facilitate the burgeoning illegal trade in puppies across the EU and increase the risk of undetected exotic disease outbreaks. Substantial public education and/or legal action is required to prevent consumers unintentionally supporting poor welfare sources of puppies.

## 1. Introduction

The number of dogs and puppies imported to the United Kingdom (UK) has increased steadily in recent years [1], with a recent peak in importations and puppy acquisitions during the COVID-19 pandemic [2]. An estimated 950,000 puppies per year are required to meet UK demand, with only 15–20% sourced from UK-based licensed breeders [3,4]. A substantial proportion of UK-owned puppies are sourced from overseas, with over 10% of UK puppies bought during the COVID-19 pandemic reported to have been acquired with a Pet Passport, which increased year-on-year between 2019–2021 [5]. Most puppies entering the UK with EU Pet Passports are sourced from, or transit through, the European Union (EU); Romania was the source of approximately half of all dog consignments imported from the EU between 2018 and 2023 (*n* = 47,887/94,543 importations comprising 69,200 dogs) [6]. This paper will explore the knowledge of UK residents about the practicalities and regulation of puppy importation, and their awareness of the risk of the exotic infectious diseases that those puppies may carry.

Legislation governing the importation of dogs has changed significantly over the past century. From 1901 to 2000, the Importation of Dogs Order (1901) required all dogs entering the UK to undergo a six-month quarantine period for rabies prevention. In 2000, this was partially replaced by the Pet Travel Scheme (PETS) following sustained pressure from the public and animal charities to adopt a system based on rabies vaccination [7] and fears that members of the public were smuggling pets into the UK to avoid expensive quarantine fees [8]. Under this version of PETS, pet dogs entering the UK from listed EU countries could avoid quarantine if they were microchipped, rabies-vaccinated at or after three months of age, tested for rabies antibodies at least six months after vaccination or after three months of age, certified by a veterinary surgeon and treated for ticks and tapeworm before entry.

In 2012, legislation was updated to align with EU Pet Passport travel rules (EU Regulation 998/2003) which shortened the required waiting period following rabies vaccination and removed compulsory tick treatment. Legislation changed again in 2014 (EU Regulation 576/2013) to introduce a minimum age of 12 weeks for rabies vaccination, mandate a 21 day wait after vaccination before travel, enhance Pet Passport security and set a maximum of five dogs per owner per trip. Non-commercial importation of dogs increased following these changes from 85,299 dogs in 2011 to 307,263 dogs in 2019 [9]. After the UK’s ”Brexit” from the EU on 1st January 2021, the PETS scheme ended, invalidating UK-issued Pet Passports. EU-issued Pet Passports remain valid for non-commercial importation of dogs into the UK [10].

Regulation governing the commercial importation of dogs is covered by the Balai directive, implemented in 2011 [11] to control the movement of puppies and dogs from listed countries for commercial reasons including sale, rescue and rehoming. This legislation, unchanged by Brexit, mandates a minimum age of importation of 15 weeks, rabies vaccination, microchipping, a health check by a veterinarian, pre-notification of importation and traceable official paperwork. Importers from some countries, including Romania, require specific “Approved Importer” status.

The UK Parliament are currently debating the Animal Welfare (Import of Dogs, Cats and Ferrets) Bill, which seeks to ban importation of puppies aged under 6 months, and bitches which are more than 42 days pregnant [12]. The current minimum legal age at which puppies can be imported to the UK via either route remains 15 weeks. Pregnant bitches can be imported up to the final week of their pregnancy, and one person can non-commercially import up to five dogs at a time, including by authorizing someone else to travel with those animals within a five-day period. Additionally, UK legislation adopted in 2020 banning third-party sales of puppies [13] does not apply to imported puppies. Evidence suggests that a significant proportion of puppies entering the UK with EU Pet Passports do not adhere to current legislation. In a recent survey, almost half (49.7%) of puppies acquired in 2021 with an EU Pet Passport were aged 7–8 weeks at sale [5]. No previous peer-reviewed studies have examined whether UK residents understand these regulations.

There are many potential risks associated with importing dogs from overseas; the substantial risks related to the welfare of those puppies and their prospective owners are discussed elsewhere [14]. Another major risk is that imported dogs may carry exotic diseases not currently endemic in the UK [15]. There is substantial evidence to support concerns about the ingress of such diseases. Systematic surveillance for exotic diseases is not performed in the UK [1,16,17], and a report from the National Audit Office (NAO) released in June 2025 [18] identifies that the UK Government’s Cabinet Office does not have a good understanding of the UK’s preparedness for an outbreak of rabies. This is despite the rabies risk increasing in some EU countries; for example, the war in Ukraine has led to a substantial increase in the number of dogs with rabies in both Ukraine and the neighboring countries, several of which are key origin countries feeding the EU puppy trade [4,19].

In addition, the NAO report [18] identifies that the UK Government do not have a plan as to how to deal with more than two concurrent exotic disease outbreaks. Multiple exotic diseases have already entered the UK with imported dogs. A 2018 investigation into the exotic disease burden in 151 imported dogs living at single UK premises (most originating from Romania) found 24% of those dogs to be carrying at least one infection with *Leishmania infantum*, *Babesia canis*, *Hepatozoon canis*, *Dirofilaria immitis*, *Brucella canis* or *Ehrlichia canis* [1]. Norman et al. [20] surveyed 3080 UK owners of imported dogs and reported that, of those dogs that had been tested, 14.8% were positive for leishmaniasis, with smaller numbers testing positive for each of *Babesia canis*, *Dirofilaria immitis*, *Ehrlichia canis*, rabies and tongueworm (*Linguata serrata*). Canine cases (which may include more than one dog) of *Brucella canis* in the UK have risen from zero in 2018 to 55 in 2022 [21]. In addition to introducing exotic diseases, imported dogs have been found to be carrying parasite vectors including *Rhipicephalus sanguineus* and *Dermacentor reticulatus* [16], which could act as domestic reservoirs for *Ehrlichia canis* and *Babesia canis*; the latter has already been documented as circulating in southeast England [22,23].

Given that surveillance is not being performed for exotic diseases, there is strong reliance on UK residents to recognize that they exist. Little research has been conducted into UK residents’ awareness of exotic disease risks posed by dogs moving into the UK from the EU. Norman et al. [20] reported that 74% of imported dog-owning respondents claimed to be aware of the differences between canine diseases present in their dog’s country of origin and the UK, but their knowledge was not tested. It might be expected that educational initiatives such as World Rabies Day [24] have led to substantial public knowledge about rabies specifically. However, an online survey of travelers to rabies-endemic countries, including UK participants, demonstrated that whilst awareness that rabies existed was high, knowledge about how to avoid transmission was poor [25]. Since that survey, substantial media attention has focused on the risks posed by *Brucella canis* following the infection of two humans in the UK [26], but it is not known how well this translates into public knowledge about the disease. Similarly, it is not clear how well people in the UK understand the risks to other dogs, and people, of exotic diseases of concern.

The aim of this study was to use an online survey to collect questionnaire data from UK residents about their awareness and knowledge of EU Pet Passports, illegal puppy importation and exotic diseases which could be carried by an imported puppy, and the risks they might pose to UK-resident dogs and people.

## 2. Materials and Methods

### 2.1. Ethical Statement

This study received ethical approval from the Social Science Research Ethical Review Board at the Royal Veterinary College (Reference: SR2023-0085). Funding for the research was from the Research England Quality-Related Strategic Priorities Fund.

### 2.2. Survey Design and Content

An online survey, entitled “Purchasing puppies survey: would you buy this puppy?” was developed to explore United Kingdom residents’ knowledge about, and attitudes towards, purchasing puppies imported from the European Union. Questionnaire development was led by Z.B., with content serially refined following discussion with R.M.A.P. and pilots with eligible respondents. The correct responses to the knowledge-based questions (see below) were checked with a European diplomate in small-animal internal medicine. The final questionnaire (appended in Supplementary Materials File S1) comprised 40 multiple-choice (MCQ) and/or free-text questions, including an optional four questions at the end for current dog owners. Logic was applied to seven questions to ensure that only those most relevant were visible to individual participants. No questions were mandatory after initial consent had been obtained. Respondents could not go back to amend previous answers once they had completed a page.

The survey was split into six sections:

1. Participants’ past and present dog ownership history and whether they were currently considering acquiring a new dog.
2. Questions based around a fictional advert for an 8-week-old Cocker spaniel puppy, exploring participants’ attitudes towards purchasing that puppy as additional information about its imported origins was iteratively revealed, and testing their knowledge about EU Pet Passports.
  Pertinent to this paper, respondents were asked the following:
  i. Whether a set of seven questions about EU Pet Passports were true or false (see Table 1 for the list of questions, their correct responses and the references used to construct the questions). Response options for each were: True; false; not sure; and don’t know. The latter two response options were included to help distinguish uncertainty from lack of knowledge. The correct answer for each question was used for statistical analysis (i.e., a binary variable of whether the respondent was correct/incorrect).
  ii. Whether they thought the 8-week-old Cocker spaniel puppy had been imported legally for sale, with response options: Yes legally imported; no, illegally imported; not sure; and don’t know. The correct response used for analysis was that they were illegally imported (the puppy was aged under 15 weeks, which is illegal via both importation routes).
  iii. To whom they believed the seller of an illegally imported puppy could be reported for action to be taken, with response options: Police; RSPCA/SSPCA/USPCA; Trading Standards; website listing the sale; local Council; UK Border Force; a veterinary practice; don’t know; and other (please specify). Again, see Table 1 for correct responses and the references used to construct the questions.
3. Questions based around a second fictional advert for a 16-week-old Jack-a-Poo (Jack Russell-cross-Poodle) imported from Romania to further explore participants’ attitudes towards imported dogs, and their awareness of the potential risks and benefits of a puppy being imported for sale to the UK to both the imported puppies themselves and their prospective owners; those data are presented elsewhere [14].
  Pertinent to this paper, respondents were also asked the following:
  i. How they thought these puppies were most likely to have travelled from Romania to the UK, with response options: Plane; car; lorry; van; don’t know; and other (please specify). Official data are not collected by the UK’s Animal and Plant Health Agency (APHA) on how animals arrive in the UK, but it is widely acknowledged that it is likely to be a vehicle rather than a plane [4,9].
4. Questions exploring respondents’ concern levels and knowledge sources related to exotic diseases potentially carried by imported dogs, and their awareness and knowledge about the transmission risks to UK-based humans and dogs from six such diseases [27]. Again, see Table 1 for the list of questions, their correct responses and the references used to construct the questions.
  Pertinent to this paper:
  i. All respondents were asked what, if any, was their level of concern about imported puppies and dogs bringing disease into the UK that are not currently found here, with response options: Not at all concerned; a little concerned; very concerned; and not something I had thought about.
  ii. Those respondents who answered the previous question expressing that they were a little or very concerned were invited to tell us where they had heard about the risk of imported diseases in puppies and dogs, with response options: Training for my job; media; social media; information campaigns from animal charities; information campaigns from UK Government/DEFRA/DAERA; own experience, e.g., travelled with or owned an imported dog; experiences of or discussion with family or friends; not sure/can’t remember; and other (please specify, free-text response).
  iii. All respondents were asked which of the following diseases they had heard of that could be associated with dogs legally and illegally imported into the UK: Rabies; brucellosis (Brucella); leishmaniasis (Leishmania); babesiosis (Babesia); ehrlichiosis (Ehrlichia); hepatazoonosis (Hepatazoon); and heartworm (Dirofilaria). For each disease, respondents could choose from response options: Yes, I have heard of it; no, I have not heard of it; and not sure. The total number of diseases each respondent who completed the question set had heard of was counted and used in statistical analysis.
  iv. For each disease a respondent had heard of, they were then asked whether they thought an infected imported puppy could infect a UK-based (a) human; and (b) dog with that disease, including via tick transmission. Response options were: Yes; no; not sure; and don’t know.
5. General demographic data, including age, nationality, gender, previous residency outside the UK and any prior history of owning or working with dogs originating in the EU, including specific job roles.
6. Optional extra questions for dog owners about the source of their most recent dog and motivations for acquiring an imported dog.

This paper focuses on reporting knowledge test questions embedded within Section 1, Section 2, Section 3 and Section 4; the attitudinal data are reported elsewhere [14]. The knowledge questions on which statistical analyses were performed in the current paper are tabulated below (Table 1), highlighting the response option used as “correct”, and the source of that information at the time of survey design. The remaining knowledge-based questions are presented in full with the relevant results.

**Table 1:** Questions presented to the respondents with their correct answers at the time of survey design, and reference sources.

Question Prefix	Question Posed	Correct Response Option(s)	Supporting Data Source(s) Used
True or false: an EU Pet Passport…	Can be issued in the UK	False	[28]
	Means that a dog or puppy can travel from the UK to European Union countries without extra paperwork	False	
	Means that a puppy or dog has been vaccinated against rabies	True	
	Means that a puppy or dog has had all their standard vaccinations	False	
	Means that a dog or puppy has tested negative for infectious diseases	False	
	Means that a puppy or dog was born in the European Union	True	
	Means that the puppy or dog is registered with a Kennel Club in the European Union	False	
Look again at the information in the advert.	Do you believe that these puppies were imported legally for sale into the UK?	No, illegally imported	[29]
Importing these puppies for sale was illegal and the seller is is breaking the law.	Who do you believe this seller could be reported to for action to be taken?	Local authority, RSPCA/SSPCA/USPCA, trading standards	[30]
n/a	How do you think these puppies were most likely to have travelled from Romania?	Overland, therefore car, lorry or van	[9]
Do you believe an infected, imported puppy could infect a human in the UK with any of these diseases (including tick transmission?	Rabies	Yes	[31]
	Brucellosis (Brucella)	Yes	[32]
	Leishmaniasis (Leishmania)	No	[33]
	Babesiosis (Babesia)	No	[34]
	Ehrlichiosis (Ehrlichia)	No	[35]
	Hepatozoonosis (Hepatozoon)	No	[36]
	Heartworm (Dirofilaria)	No	[37]
Do you believe an infected, imported puppy could infect another dog in the UK with any of these diseases (including tick transmission?)	Rabies	Yes	[38]
	Brucellosis (Brucella)	Yes	[39]
	Leishmaniasis (Leishmania)	Yes	[40]
	Babesiosis (Babesia)	Yes	[23]
	Ehrlichiosis (Ehrlichia)	Yes	[35]
	Hepatozoonosis (Hepatozoon)	Yes	[41]
	Heartworm (Dirofilaria)	No	[37]

### 2.3. Participant Recruitment

The survey was hosted on SurveyMonkey and disseminated via snowball sampling using social media (Facebook, Twitter, TikTok, Instagram) between 29th May and 30th June 2023. Details of the study were also shared by a wide range of individuals, charities and dog/veterinary-related organizations via their social media and/or email lists (see acknowledgments for details). In addition, direct emails were sent to owners who had bought a dog from the Pets4Homes website in the previous calendar year and respondents to The Royal Veterinary College Pandemic Puppies team’s previous surveys [2,42] who had consented to be contacted about additional research opportunities.

Given the iterative nature of the survey, which released more information about the imported nature of the fictional puppies as questions progressed, we elected to advertise the survey by stating that we were interested in understanding how the information in puppy adverts affects puppy buying intentions. This strategy aimed to avoid biasing our respondent population towards people with a pre-formed viewpoint and/or experiences regarding imported dog health and welfare.

### 2.4. Data Cleaning

Raw data from SurveyMonkey was exported into Microsoft Excel (Windows 2016) for manual cleaning. Responses where repeat IP addresses were linked to identical answers were removed, as were responses from non-eligible participants, and responses where no data was inputted after the initial demographics section. Responses with low volumes or where responses were related (e.g., responses ”Don’t know” and ”Not sure”) were grouped; year of most recent dog acquisition was also grouped. In addition, different types of missing values (skipped, unanswered, not applicable) were manually distinguished. Free-text responses were back-coded to the relevant multiple-choice answer where possible or collated in new categories using qualitative content analysis [43].

### 2.5. Statistical Analysis

After initial cleaning, descriptive statistics (frequency and percentages) were calculated for all variables. Four overall scores were calculated per relevant participant: the sum of correct answers to the questions related to EU Pet Passports (possible score range 0–7); the sum of the number of exotic diseases of which each respondent was aware (possible score range 0–7); the sum of correct answers to whether those diseases could infect a UK-based human (possible score range 0–7); and the sum of correct answers as to whether those disease could infect a UK-based dog (possible score range 0–7). The number of valid respondents to questions relating to EU Pet Passports, and the sum of exotic diseases of which respondents were aware, were then refined to remove any respondents from follow-on analyses who had not answered all seven questions in each question set. Potential respondent demographic variables (current, prior and anticipated dog ownership; whether they had lived overseas; history of working with EU dogs in different roles; history of owning a dog from overseas) were collapsed into binary variables. Respondent age and year of acquisition of their most recent dog were collapsed into aggregated groups to ensure sufficient group size for analyses. Descriptive data are reported with the mean and standard deviation if normally distributed, and with the median and interquartile range if not normally distributed.

Statistical analysis was conducted using the open-source software Jasper’s Amazing Stats Package (JASP; version 0.19.3.0). Data distribution was assessed using visual inspection of histograms. Variables for which the smallest subgroup contained at least 10% of the overall population for that question were taken forward for univariable linear regression to determine whether they predicted the total number of correctly answered questions about EU Pet Passports, and the number of diseases respondents had heard of.

Multivariable linear regression analysis was used for risk factor analysis. Multicollinearity was assessed using collinearity diagnostics and inspection of standard errors for inflation. Model building used variables liberally associated with the outcome (*p* < 0.2) in the univariable analysis; these were taken forwards, and their retention was assessed using backwards stepwise elimination. The quality of the model’s fit was assessed using Q-Q plots for standardized residuals, residual histograms to assess for normality, collinearity tolerance tests and VIF. Model fit quality was evaluated using the adjusted R-squared approach. Descriptive data are reported with the mean and standard deviation (SD) if normally distributed, and with the median and interquartile (IQR) range if not normally distributed. Statistical significance was set at 5% (*p* < 0.05).

## 3. Results

The survey collected 7746 responses. Cleaning removed 562 responses, comprising repeat IP addresses with identical responses (*n* = 4); ineligible respondents (non-UK residents, *n* = 16; under 18 years of age, *n* = 2); and responses blank after the initial demographic data questions (*n* = 540). This left *n* = 7184 unique, valid responses for analysis. No questions were mandatory, therefore each had a different response rate; the sample size for individual questions is provided with each set of results.

### 3.1. Participant Demographics

Most respondents who provided demographic information were female (*n* = 4894/5323; 91.9%). Respondents populated all eligible age groups, with 25–34-year-olds the most represented (*n* = 1130/5336; 21.2%), closely followed by 35–44-year-olds (1067; 20.0%) and 45–54-year-olds (1059; 19.8%). As anticipated based on the UK population, most respondents lived in England (*n* = 3863/5322; 72.6%), with residents of Scotland contributing 1146 (21.6%) responses, Welsh residents providing 258 (4.9%) responses, and Northern Irish residents relatively under-represented with 53 (1.0%) responses. Just under 8.0% of respondents (7.9%, *n* = 422/5317) had previously been a permanent resident of a country currently in the European Union, with 7.1% (*n* = 380/5317) having resided in a country not currently within the European Union.

#### 3.1.1. Dog Ownership Experience

Current dog owners accounted for most respondents (*n* = 5932/7184; 82.6%), with only *n* = 487/7184 (6.8%) respondents having never owned a dog. Of the those who had previously, or currently, owned a dog, 57.8% of respondents (*n* = 3835/6651) obtained their most recent dog between 2020 and 2023 (range: pre-1995–2023), with almost three-quarters of the dogs (*n* = 4801/6690; 71.7%) being acquired as a puppy aged under 16 weeks of age. When questioned about their future dog-buying intentions, around one-quarter (*n* = 1733/7184, 24.1%) of respondents were currently considering, actively looking for, or in the process of, purchasing a dog or puppy. This included *n* = 212/488 (43.4%) of the respondents who had never previously owned a dog. Of respondents who gave details about whether they had ever travelled with a dog outside the UK, *n* = 560/5329 (10.5%) responded affirmatively. The majority (*n* = 394/560; 70%) of those owners had previously resided in a country outside the United Kingdom.

Most dog owners (*n* = 3531/4119; 85.7%) who completed the optional questions in section five about dog ownership had not owned a dog imported from outside the UK. Of those *n* = 588 owners who had owned at least one dog born outside the UK, *n* = 364 (61.9%) had rescued/adopted at their dog(s) from abroad, *n* = 170 (28.9%) had purchased their dog(s) from abroad, *n* = 82 (13.9%) had fostered one or more dogs born outside the UK and a further *n* = 42 (7.1%) were not sure or didn’t know if their dog had been born outside the UK.

#### 3.1.2. Respondents’ Experience Working with Dogs That Travel to or from the European Union

Of the *n* = 5323 respondents to the question asking whether they had ever worked with dogs travelling to or from the European Union, *n* = 1132 (21.3%) responded that they had occupied one or more relevant roles. Of these, 30.5% (*n* = 345/1132) had worked as veterinary surgeons; 28.6% (*n* = 324/1132) as veterinary nurses; 17.1% (*n* = 194/1132) had been in other veterinary practice-based roles; 13.1% (*n* = 149/1132) had worked in shelters which took dogs from the European Union; 11.0% (*n* = 125/1132) had worked as a dog trainer; 9.1% (*n* = 104/1132) had shown dogs either in Europe or dogs that had been bred in the EU; 4.6% (*n* = 52/1132) had worked in boarding kennels; 3.6% (*n* = 41/1132) had fostered dogs from the EU; 1.1% (*n* = 13/1132) had worked with dogs in the army; 1.1% (*n* = 12/1132) had worked as couriers; 0.7% (*n* = 8/1132) had occupied security roles; 0.7% (*n* = 8/1132) had worked in border control; 0.4% (*n* = 5/1132) had worked in a search and rescue roles and 6.4% (*n* = 73/1132) had occupied other unspecified but relevant roles.

### 3.2. Respondents’ Concerns About Imported Diseases

When asked whether they were concerned about new diseases being introduced to the UK by a puppy imported from overseas, *n* = 110/5452 (2.0%) of respondents were not at all concerned, *n* = 1479/5452 (27.1%) were a little concerned, *n* = 3375/5452 (61.9%) were very concerned and *n* = 491/5452 (9.0%) had not thought about this topic before. Of those respondents who expressed no concern, *n* = 93/110 (84.5%) were current dog owners, as were *n* = 394/491 (80.2%) of those who had not previously thought about this topic. Of the *n* = 369 respondents who had never owned a dog and answered this question, *n* = 218 (59.0%) were very concerned, and *n* = 100 (27.1%) a little concerned, with only *n* = 6 (1.6%) unconcerned and *n* = 45 (12.2%) who had not thought about this topic.

#### Respondents’ Information Sources About Imported Diseases

The *n* = 4851 respondents who answered that they were a little or very concerned about imported diseases were asked to choose the places they had heard about this topic from a multiple-choice list, with the option to add additional free-text responses. Of the *n* = 4554/4851 (93.8%) respondents who completed the list, the most common information sources listed were information campaigns from animal charities (*n* = 2019; 44%) and social media posts (*n* = 1879; 42%; see Table 2). When analyzed by ownership category, the same information sources were also the most recalled by current dog owners. For previous dog owners, information from charities was still the top information source, with similar respondent numbers also recalling seeing information in the media and social media. However, for respondents who had never owned a dog, training for their job was the most common information source, followed by the media and social media. The median number of places current, previous and non-dog owners recalled seeing information was 2 (IQR 1–3).

Free-text responses were provided by *n* = 397 people, the majority of which included details expanding on the multiple-choice options they had chosen. The most common additional information source provided in the free text was their own veterinary surgeon or a veterinary surgeon whose online posts they had read (*n* = 59). A further *n* = 54 people said in the free text that they did not know about this topic before completing the survey but now had some level of concern about it.

### 3.3. Responses to Knowledge Test Questions

#### 3.3.1. True/False Questions About EU Pet Passports

At least one of the seven questions about EU Pet Passports was answered by *n* = 6733 respondents; each question had a slightly different response rate (see Table 3), with *n* = 6655 answering all seven questions. The median number of correct answers was 3 (IQR 1–4). For each question, at least 34.0% of respondents were either not sure or did not know. The question eliciting the greatest uncertainty, and the greatest number of incorrect answers, was whether an EU passport means that the puppy or dog was born in the EU. The question eliciting the highest percentage of correct answers was whether an EU Pet Passport meant that the dog was registered with a European Kennel Club.

#### 3.3.2. Risk Factor Analysis for the Number of Questions About Pet Passports Answered Correctly

After removal of all respondents who had not answered all seven of the above questions, *n* = 6655 respondents’ scores were eligible for linear regression analysis. Twelve of the 17 variables assessed using univariable linear regression for their association with the outcome measure “number of EU Pet Passport questions answered correctly” were liberally associated (*p* < 0.2; see Table S1). Following multivariable modelling, five variables were retained in the final model, all of which were significant (Table 4). The adjusted R-squared analysis indicated acceptable model fit (0.146, *p* < 0.001). Having taken a dog abroad (compared to having never taken a dog abroad), ownership of a dog born outside the UK (compared to not having owned a dog born outside the UK), and having worked as a veterinary surgeon or a veterinary nurse with dogs originating from the EU (compared to having not had these roles) were significantly associated with correctly answering more questions. Having worked with EU dogs in a shelter setting (compared to not having had this role) was significantly associated with answering more questions incorrectly.

#### 3.3.3. Is It Legal to Import an 8-Week-Old Puppy to the UK?

Responses regarding whether respondents thought the 8-week-old Cocker spaniel puppies included in Section 2 of the survey had been legally or imported were collected from *n* = 6514 people. Of these, *n* = 3169 (48.6%) correctly answered that the puppies had been illegally imported, *n* = 470 (7.2%) incorrectly answered that the puppies were legally imported, *n* = 2105 (32.3%) people were not sure and *n* = 770 (11.8%) responded that they did not know.

#### 3.3.4. To Whom Could an Illegal Seller Be Reported?

Responses were collected from *n* = 6283 people, with most supplying multiple answers and *n* = 193 respondents adding free text. The most selected multiple-choice responses were the Police, UK Border Force and RSPCA/SSPCA (see Table 5). Additional options were added from the free text: DEFRA/APHA (*n* = 94), Kennel Club/Breed groups (*n* = 18), and HMRC (*n* = 13). An additional *n* = 27 commented that they did not think anything would be done even if it was reported. Other free-text responses included social media or local press (*n* = 8), the seller’s local member of parliament (*n* = 5), and the dog warden (*n* = 4).

#### 3.3.5. Via What Route Would Puppies Most Likely Have Been Imported from Romania to the UK?

Responses to the question asking how the puppies might have arrived in the UK were collected from *n* = 5964 people. The most common answer, chosen by over 51.6% (*n* = 3077) respondents, was that the dogs were transported via a van, followed by a lorry (17.2%; *n* = 1030), plane (8.0%; *n* = 502) and car (5.5% (*n* = 326). A further *n* = 997 (16.7%) said they did not know. Additional response options of a boat (*n* = 7) and more than one of the above (*n* = 25) were created from the free text. Of the *n* = 91 free-text responses, *n* = 28 comments related to the respondent’s discomfort about thinking about these puppies being transported at all; the others either further explained their MCQ responses or added additional options.

#### 3.3.6. Imported Disease Awareness

Most of the *n* = 5415 respondents who answered at least one question about whether they had heard of a list of imported diseases had heard of rabies (99.2%), heartworm (85.0%) and brucellosis (64.9%), and over half had heard of leishmaniasis (54.0%). However, only one-third had heard of babesiosis (34.2%), and only around a quarter had heard of ehrlichiosis (26.7%) and hepatozoonosis (23.1%; see Table 6).

Of *n* = 5231 respondents who answered all the questions, the median number of the individual diseases they recalled having heard of was 4 (IQR 2–5); *n* = 740 (14.1%) respondents had heard of all seven diseases. Of *n* = 19 respondents who recalled having heard of none of the diseases, all were current or previous dog owners and three had previously lived in the European Union. Of the *n* = 508 respondents who had heard of only one disease *n* = 499 (98.2%) had heard only of rabies; *n* = 8 (1.6%) had heard only of heartworm and *n* = 1 had heard only of Brucellosis. Of *n* = 1126 respondents who had heard of two diseases, *n* = 110 (97.6%) had heard of rabies, *n* = 908 (80.6%) had heard of heartworm, *n* = 159 (14.1%) had heard of brucellosis, *n* = 15 (1.3%) had heard of leishmaniasis, *n* = 6 (0.5%) had heard of hepatozoonosis, *n* = 4 (0.4%) had heard of babesiosis and none had heard of ehrlichiosis.

#### 3.3.7. Risk Factor Analysis for the Number of Diseases Respondents Had Heard Of

After removal of all respondents who had not answered all seven of the above questions, *n* = 5231 respondents’ scores were eligible for analysis in a linear regression. Fifteen of the 17 variables assessed using univariable linear regression for their association with the outcome measure “number of diseases respondents had heard of” were liberally associated (*p* < 0.2; see Table S2). Following multivariable modelling, six variables were retained in the final model (Table 7). The adjusted R-squared analysis indicated acceptable model fit (0.326, *p* < 0.001). Of the six variables, only respondent gender (being female compared to male), being aged 55–64 (compared to being aged 35–44) and having worked with EU dogs as a veterinary surgeon (compared to not having had this role) were predictive of having heard of more diseases. Being aged 18–24 (compared to 35–44) was a significant predictor of having heard of fewer diseases.

#### 3.3.8. Knowledge About Transmission Routes and Risks to Human from an Infected Puppy

Participants who had heard of the diseases above were asked two further questions about the risks posed to people from an imported puppy infected with that disease. Table 8 shows the responses per question; overall, *n* = 5318 respondents answered questions about at least one disease. The median number of correct answers was 1 (IQR 1–2); the range of correct answers was 0/1–7/7. Of the *n* = 508 respondents who had only heard of one disease, *n* = 340 (67.0%) answered correctly for that disease. Of *n* = 740 respondents who had heard of all seven diseases, only *n* = 58/710 (8.1%) who provided responses to all these questions correctly answered all seven questions about the risks of dog–human transmission; the median number of correct answers in this subpopulation who had heard of all the diseases was 3 (IQR 2–5).

Rabies was the disease for which the most respondents provided the correct answer, though *n* = 571/5264 (10.8%) respondents were either incorrect or uncertain as to whether rabies could infect people. Hepatazoonosis was the disease which the most respondents (*n* = 459/1182; 38.8%) incorrectly thought could infect humans; brucellosis was the disease which the most respondents (*n* = 118/3410; 3.5%) incorrectly thought could not infect humans. The question eliciting the greatest uncertainty was heartworm, with *n* = 2578/4502 (57.1%) respondents expressing uncertainty as to whether it could be zoonotic; the overall number of respondents who expressed uncertainty was >40% for all other diseases apart from rabies.

#### 3.3.9. Knowledge About Transmission Routes and Risks to Another Dog from an Infected Puppy

Respondents appeared more confident about their responses in relation to an infection with diseases that they had heard of being transmitted to a dog. Table 9 shows the responses per question; overall, *n* = 5316 respondents answered questions about one disease. The median number of correct answers was 3 (IQR 1–3); the number of correct answers again ranged from 0/1 to 7/7. Of the *n* = 508 respondents who had only heard of one disease, *n* = 453 (89.2%) answered correctly for that disease. Of the *n* = 740 respondents who had heard of all seven diseases, only *n* = 30 (4.1%) correctly answered all seven questions about the risks of dog–dog transmission; the median number of correct answers in this subpopulation who had heard of all seven diseases was 1 (IQR 1–2).

Rabies was again the disease for which the most respondents provided the correct answer, with only 3.7% respondents (*n* = 195/5256) responding either incorrectly or with uncertainty. Heartworm was the disease which the most respondents (*n* = 2968/4508; 65.8%) incorrectly thought could infect another dog based in the UK; leishmaniasis was the diseases which the most respondents (*n* = 322/2843; 11.7%) incorrectly thought could not infect another dog based in the UK. The question eliciting the greatest uncertainty was again heartworm, with *n* = 274/4508 (24.1%) respondents unsure of the correct answer; overall uncertainty was lower for all questions than for the corresponding question about risks to humans.

Only one respondent to the survey correctly answered all fourteen questions about risks to humans and dogs; that person incorrectly believed that an EU Pet Passport meant that a dog had been tested for infectious diseases.

## 4. Discussion

This large-scale survey suggests that UK residents have limited understanding of the legal, logistical, or canine and human disease risks related to importing puppies from the European Union. Given the documented surge in dogs being imported to the UK, our findings suggest that UK public knowledge needs to be substantially bolstered to protect canine health and welfare and public health. With some respondents not even being confident that they had heard of rabies, despite widespread messaging regarding this disease, there is substantial work to do to ensure that the UK canine and human populations remain safe in the face of substantial welfare, and emerging disease threats.

We identified substantial knowledge gaps relating to EU Pet Passports. For example, only 30.8% of respondents correctly identified that an EU Pet Passport could no longer be issued in the UK, and only 24.1% were confident that a dog with an EU Pet Passport had been born in the EU. Furthermore, only 48.6% of respondents correctly identified that an 8-week-old puppy born in the European Union must have been illegally imported. This suggests that even if an underage puppy is knowingly sold with an EU Pet Passport, many prospective owners will not automatically identify that puppy as either having been bred overseas or illegally sold. Legal age at acquisition appears to be a general source of confusion to UK dog owners, with previous reports that one in four puppies in the UK are acquired under the legal age (for domestic sales) of 8 weeks [44]. Further sources of incorrect responses included participants believing that puppies sold with EU Pet Passports were vaccinated to UK standards (38.8%), had been tested negative for infectious diseases (45.9%) or not vaccinated against rabies (16.8%). These false beliefs may provide reassurance to owners who find out their prospective puppy has a passport (e.g., during communication with their seller) or upon sale, and lead to lowered concern over acquisition from overseas (and consequent increased likelihood of sales going ahead) and insufficient provision of preventative care. Unexpectedly, the most common false belief regarding passports was that a puppy being sold with an EU Pet Passport meant that a puppy or dog was registered with a European kennel club; this may be attractive to many owners, given the desirability of purebred dogs by some owners [45,46,47].

Correctly answering more questions about EU Pet Passports was statistically associated with having worked with or owned a dog from abroad or having personally travelled abroad with a dog. This suggests that information about the current legal status of EU Pet Passports does not reach even the wider UK dog-owning population. This lack of knowledge will only serve to embolden those involved in the illegal puppy trade, which is burgeoning across the EU [4]. Respondents had better knowledge about where to report illegal sellers, perhaps testament to recent campaigning work by Dogs Trust and RSPCA [9,30]. Similarly, most respondents correctly answered that the puppies were likely to have travelled overland from Romania, but it was worrying that 8% of respondents, over *n* = 500 individuals, thought they were most likely to have travelled by plane. This perception may lead to a substantial underestimate of the welfare detriment to those puppies due to their arduous overland journey; this is discussed further elsewhere.

When asked about where they had heard about the infectious disease risks associated with imported dogs, the most common information sources listed by respondents were the media, social media and animal charities. It was notable that, when tested, respondents knew significantly more about disease risks to dogs than to humans, which may suggest that exotic diseases are not the focus of information sources accessed. Ophorst et al. [48] identified that the Dutch media discussed zoonotic disease just twice in 2855 articles about dogs published over a ten-year period; a similar study has not been performed in the UK, and it is not known how much animal charities focus education on exotic or zoonotic diseases. Stakeholder attitudes towards providing education on exotic and zoonotic disease in the UK would be a valuable topic for future research.

We omitted veterinary surgeons as a response option when asking respondents for disease information sources. Given that pet owners commonly state a preference to receive animal health information from a veterinary surgeon [49,50,51], it might have been anticipated that more than *n* = 59 respondents would manually add them as an information source, given the high percentage of respondents who were current dog owners. UK veterinary surgeons may not feel it relevant to discuss exotic diseases with owners or may not have the time during busy preventive healthcare consultations [52]. In addition, whilst the One Health movement has been widely promoted [53,54], UK veterinary surgeons may still not see themselves as having a role in educating owners about the human risks of potential zoonoses. Previous research has identified that veterinary surgeons rarely make recommendations to owners about which websites to access [50]; this may magnify the problem. Our study suggests that barriers amongst UK veterinary surgeons to providing information to pet owners about exotic and zoonotic disease risks should be explored.

Whilst 89% of respondents expressed concern about risks associated with exotic diseases, this level of concern was not matched by their awareness of specific diseases. Fewer than half recalled having ever heard of each of *Babesia* spp., *Ehrlichia* spp., *Hepatozoon* spp. or dirofilarial heartworm. As expected, veterinary surgeons who had worked with EU dogs were the respondent demographic who had heard of more of the diseases than any other group. However, it was surprising that veterinary nurses did not have a differentially higher level of disease recognition than other groups. Veterinary nurses have previously been described as having a “vital role” in educating clients about imported disease risks and formulating parasite control plans [55]; greater education at the undergraduate level or continuing professional development for this professional group may be required to confidently fulfil this role. It was also unexpected that amongst respondents who had heard of only two diseases, dirofilarial heartworm would be far more widely recognized than *Brucella canis*, given the widely publicized case of a UK resident becoming infected with brucellosis by their imported dogs 10 months before survey launch [56]. This poor level of disease awareness should be of substantial concern, given the increasing threat of these diseases becoming endemic in the UK [15] and the UK Government’s implicit reliance [18] on residents as the first line in disease surveillance.

Even where participants had heard of diseases, knowledge about the transmission risks related to those diseases was typically poor, with only one respondent currently answering both question sets, suggesting that even veterinary surgeons do not fully understand the risks. Our findings build on those of Norman et al. [20]. Whilst 74% of UK-based imported dog owners in that survey believed they were aware of differences between diseases in the UK and their dog’s country of origin, many expressed uncertainties about which diseases their dog had been tested for. Norman et al. [20] did not find that knowledge about testing for any single disease was differentially better. Our respondents displayed better knowledge about rabies than other diseases, with 89.2% of respondents correctly answering that UK-based humans were at risk from an infected dog, and 96.3% correctly understanding its dog–dog transmission risk. This knowledge could be explained by rabies being the only disease included in our research which was historically endemic in the UK [38], leading to widespread public discourse [57], awareness-raising campaigns [24] and specific legislation [58,59]. It is therefore likely that the UK public have been passively exposed to information about rabies, but not the other diseases included.

Our results clearly point to the need for improved education of UK residents about the legalities and disease risks associated with importing dogs from the EU; the question is how best to achieve that. Three factors have recently been proposed as underlying whether people seek, or actively avoid, specific pieces of information [60]. These are action (whether the information will help, hinder or have no effect on the individual’s ability to avoid harm and increase reward); affect (what, if any, impact the information will have on an individual’s feelings); and cognition (whether the information will improve an individual’s ability to comprehend and anticipate their reality). Those authors also highlight the impact of cognitive biases such as illusions of knowledge and overconfidence biases in reducing information seeking in certain populations. Exploring these factors may be beneficial to better understand motivators and barriers to accessing, and sharing, information related to imported puppies and exotic diseases. The success of education campaigns run by animal charities is rarely evaluated [61]. Given their reported importance as an education source about exotic disease risks, closing that feedback loop would likely be a useful step in understanding how best to educate the public.

This study had several strengths, and some limitations. This is the largest survey conducted to date assessing the UK public’s knowledge of canine imported diseases, and we incorporated both dog owners and non-owners (including those seeking to own a dog in the future) from a wide demographic range. With over one-fifth of respondents having a dog-related job role, of which approaching three-quarters were veterinary-practice based, we acknowledge that our respondents may have a different level of education on the topics compared to the baseline the UK population; however, this serves only to make our findings more concerning. The high percentage of female respondents is in line with our previous survey work and that of others [42,62], and is of benefit, given that most canine care appears to be provided by women in heterosexual households [63]. The survey was disseminated online, which may have excluded some participants, but recent data suggest that 98% of UK residents now have internet access [64], so the risk of substantial sampling bias is low. This study preceded the rise in artificial intelligence as an accessible source of knowledge; it would be fascinating to know whether that is now an influential, and accurate, information source for UK residents on pet health.

Our use of survey logic permitted a bespoke user experience and allowed us to ensure that only participants who had heard of specific diseases were asked further questions about them, which should have reduced the amount of blind guessing. However, it would have been useful to include those respondents who were not concerned about imported diseases in our survey of information sources to determine whether they were seeing the same information. It would also have been interesting to re-survey all respondents’ level of concern about imported diseases at the end of the survey, particularly given that several respondents commented within the survey that it had been educational. Fifty-three respondents were based in Northern Ireland, where some aspects of dog movement legislation differ from that of the UK mainland; we are unable to determine whether this impacted those respondents’ answers due to the limited sample size. The number of respondents who had lived in a non-EU country was greater than expected, and could possibly have been bolstered by UK residents recognizing the UK as now outside the EU.

Data about public knowledge relating to parasites such as *Lingata serrata*, *Echinococcus multilocularis* and *Thelazia callepaedia* were not gathered for brevity, but we did include the top four exotic diseases of concern recently identified by stakeholder analysis [17]. *Hepatozoon* spp. has not yet been recognized to have been transmitted from dog to dog within the UK, but autochtonous transmission is possible, given that the disease has already been detected in three imported dogs [41], and the vector *Rhipicephalus sanguinensis* is already present in the UK [65]. The substantial difference in the number of respondents correctly answering human versus canine disease risks may in part have been down to an order effect or confirmation bias, as “yes” was the correct answer to all but one of the human zoonotic questions, but only two of the canine ones. However, the percentage of respondents who were unsure of the answer was substantially higher for most questions relating to dog–human transmission that for dog–dog transmission, so it appears that we did identify a genuine difference in knowledge.

## 5. Conclusions

The knowledge of UK residents about the existence of, and risks posed by, dog-carried exotic disease is currently limited. This requires urgent improvement to safeguard the health of UK-resident human and canine populations. Knowledge deficits regarding the mechanisms by which dogs are imported into the UK and legality of sales are also widespread and may facilitate the burgeoning illegal trade in puppies across the EU. Further public education and/or legal action are needed to prevent consumers unintentionally supporting this poor-welfare source of puppies.

## Figures and Tables

**Table 2:** Information sources from which respondents recalled having learnt about imported diseases. Respondents were able to choose all answers which applied, so the total number of responses exceeds the number of respondents.

Information Source	Total Number of Respondents Who Had Learnt from This Information Source (*n* = 4554) *n* (%)	Current Dog Owning Respondents Who Had Learnt from This Information Source (*n* = 3831) *n* (%)	Respondents Who Had Never Owned a Dog and Had Learnt from This Source (*n* = 278) *n* (%)	Respondents Who Had Previously Owned a Dog and Had Learnt from This Source (*n* = 445) *n* (%)
Information campaigns from charities	2019 (44.3%)	1732 (45.2%)	92 (33.1%)	195 (43.8%)
Social media	1879 (41.3%)	1609 (42.0%)	100 (36.0%)	170 (38.2%)
Media	1786 (39.2%)	1518 (39.6%)	96 (34.5%)	172 (38.7%)
Experiences of friends and family	1214 (26.7%)	1038 (27.1%)	61 (16.5%)	115 (25.8%)
Training for job role	1157 (25.4%)	898 (23.4%)	127 (45.7%)	132 (29.7%)
Information from UK Government e.g., DEFRA, DAERA	1009 (22.2%)	882 (23.0%)	45 (16.2%)	82 (18.4%)
Own experience (e.g., travel with family dog)	506 (11.1%)	471 (12.3%)	8 (2.9%)	27 (6.1%)
Not sure/can’t remember	570 (12.5%)	452 (11.8%)	46 (16.5%)	72 (16.2%)
Total	10,140	8600	575	965

**Table 3:** Responses to a set of seven questions testing respondents’ knowledge about EU Pet Passports. The stem of the question posed was: “True or false:…” Correct responses for each question are highlighted in bold.

Question Posed (Total Number of Respondents)	Number of Responses (%)			
	True	False	Not Sure	Don’t Know
An EU Pet Passport can be issued in the UK (*n* = 6703)	1767 (26.4%)	**2063 (30.8%**)	1566 (23.4%)	1307 (19.5%)
An EU Pet Passport means that a puppy or dog can travel from the UK to European countries without extra paperwork (*n* = 6720)	**2169 (32.2%)**	1911 (28.4%)	1640 (24.2%)	1000 (14.9%)
An EU Pet Passport means that a puppy or dog has been vaccinated against rabies (*n* = 6713)	**2989 (44.5%)**	1125 (16.8%)	1441 (21.5%)	1558 (23.2%)
An EU Pet Passport means that a puppy or dog has had all their standard UK vaccinations (*n* = 6715)	1573 (23.4%)	**2604 (38.8%)**	1414 (21.1%)	1124 (16.7%)
An EU Pet Passport means that the dog or puppy has tested negative for infectious diseases (*n* = 6704)	876 (13.1%)	**3077 (45.9%)**	1539 (23.0%)	1212 (18.1%)
An EU Pet Passport means that a puppy or dog was born in the European Union (*n* = 6717)	**1617 (24.1%)**	2072 (30.1%)	1833 (27.3%)	1195 (17.8%)
An EU Pet Passport means that a puppy or dog is registered with a European kennel club (*n* = 6708)	109 (1.6%)	**4279 (63.8%)**	1103 (16.4%)	1217 (18.1%)

**Table 4:** Final multivariable linear regression model for the total number of Pet Passport questions answered correctly (*n* = 6655). • Coefficient. * 95% confidence interval. Significant results are formatted bold.

Variable	Category	B•	Std. Error	T	95% CI *	*p* Value
Respondent has previously taken a dog abroad	**Yes**	**0.563**	**0.172**	**3.277**	**0.226–0.901**	**0.001**
	No	Reference				
Respondent has owned a dog born outside the UK	**Yes**	**0.338**	**0.154**	**2.193**	**0.035–0.640**	**0.029**
	No	Reference				
Respondent has worked with EU dogs as a veterinary surgeon	**Yes**	**1.394**	**0.140**	**9.969**	**1.119–1.668**	**<0.001**
	No	Reference				
Respondent has worked with EU dogs as a veterinary nurse	**Yes**	**0.812**	**0.135**	**6.012**	**0.547–1.078**	**<0.001**
	No	Reference				
Respondent has worked with EU dogs in a shelter setting	**Yes**	**−0.469**	**0.162**	**−2.904**	**−0.787–−0.152**	**0.004**
	No	Reference				

**Table 5:** Responses (*n* = 6283) to the question “Who do you believe an illegal seller could be reported to? Tick all that apply”. * denotes option added from the free-text responses (all options with >10 respondents tabulated). Correct answers are highlighted in bold.

Organization/Person to Whom This Could Be Reported	Number of Responses (%)
**RSPCA/SSPCA**	**4457 (70.9%)**
Police	4101 (65.3%)
**Trading standards**	**3733 (59.4%)**
UK Border Force	3650 (58.1%)
Website listing the sale	3351 (53.3%)
**Local council**	**2794 (44.5%)**
A veterinary practice	1162 (18.5%)
Don’t know	325 (5.2%)
* DEFRA/APHA	94 (1.5%)
* No point reporting it, nothing will be done	27 (0.4%)
* Kennel Club/Breed groups	18 (0.3%)

**Table 6:** Responses to a multiple-choice question asking whether participants had heard of a list of diseases. The causal agent in brackets below was presented to respondents. The number of respondents to questions about whether they had heard of the diseases listed ranged from *n* = 5348 to *n* = 5395.

Disease (No of Respondents)	Yes, I’ve Heard of It *n* (%)	No, I’ve Not Heard of It *n* (%)	Not Sure If I’ve Heard of It or Not *n* (%)
Rabies (*n* = 5392)	5350 (99.2%)	25 (0.5%)	17 (0.3%)
Brucellosis (Brucella; *n* = 5374)	3490 (64.9%)	1656 (30.8%)	228 (4.2%)
Leishmaniasis (Leishmania; *n* = 5381)	2908 (54.0%)	2277 (42.3%)	196 (3.6%)
Babesiosis (Babesia; *n* = 5351)	1828 (34.2%)	3250 (60.7%)	273 (5.1%)
Ehrlichiosis (Ehrlichia; *n* = 5348)	1427 (26.7%)	3605 (67.4%)	316 (6.0%)
Hepatozoonosis (Hepatozoon; *n* = 5354)	1235 (23.1%)	3709 (69.3%)	410 (7.7%)
Heartworm (Dirofiliaria; *n* = 5395)	4587 (85.0%)	707 (13.0%)	101 (1.9%)

**Table 7:** Final multivariable linear regression model for the total number of diseases individual respondents had heard of (*n* = 5231). • Coefficient. * 95% confidence interval. Significant results are formatted bold.

Variable	Category	B•	Std. Error	T	95% CI *	*p* Value
Respondent gender	**Female**	**0.984**	**0.425**	**2.315**	**0.140–1.829**	**0.023**
	Male	Reference				
Respondent age	**18–24**	**−0.719**	**0.377**	**−1.907**	**−1.467–0.030**	**0.060**
	25–34	0.110	0.288	0.380	−0.463–0.682	0.705
	35–44		Reference			
	45–54	−0.095	0.370	−0.257	−0.829–0.639	0.605
	**55–64**	**1.005**	**0.478**	**2.104**	**0.057–1.953**	**0.038**
	65+	−0.456	0.519	−0.887	−1.487–0.576	0.383
Respondent has ever owned a dog	Yes	−0.466	0.354	−1.314	−1.170–0.238	0.192
	No	Reference				
Respondent has owned a dog born outside the UK	Yes	−0.221	0.426	−0.519	−1.068–0.626	0.605
	No	Reference				
Respondent was previously an EU resident	Yes	0.714	0.363	1.969	−0.006–1.434	0.052
	No	Reference				
Respondent has worked with EU dogs as a veterinary surgeon	**Yes**	**0.999**	**0.232**	**4.303**	**0.538–1.460**	**<0.001**
	No	Reference				

**Table 8:** Responses to the question “Do you believe an infected, imported puppy could infect a human in the UK with any of these diseases (including tick transmission)?” Note that the widely varying response rate is due to the individual diseases only being visible to those participants who had responded that they had heard of that disease in the previous question. Correct answers are highlighted in bold.

Disease (Number of Respondents per Question of Those Who Heard of the Disease	Yes, Infected Puppy Could Infect Human *n* (%)	No, Infected Puppy Could Not Infect Human *n* (%)	Not Sure *n* (%)	Don’t Know *n* (%)
Rabies (*n* = 5264)	**4693 (89.2%)**	70 (1.3%)	404 (7.08%)	97 (1.8%)
Brucellosis (Brucella; *n* = 3410)	**1904 (55.8%)**	118 (3.5%)	1040 (30.4%)	348 (10.2%)
Leishmaniasis (Leishmania; *n* = 2841)	1047 (36.9%)	**596 (21.0%)**	943 (33.2%)	255 (9.0%)
Babesiosis (Babesia; *n* = 1768)	574 (32.5%)	**331 (18.7%)**	729 (41.2%)	134 (7.6%)
Ehrlichiosis (Ehrlichia; *n* = 1376)	361 (26.2%)	**296 (21.5%)**	613 (44.5%)	106 (7.7%)
Hepatazoonosis (Hepatazoon; *n* = 1182)	459 (38.8%)	**197 (16.7%)**	448 (37.9%)	78 (6.6%)
Heartworm (Dirofiliaria; *n* = 4502)	698 (15.5%)	**1226 (27.2%)**	1988 (44.1%)	590 (13.1%)

**Table 9:** Responses to the question “Do you believe an infected, imported puppy could infect another dog in the UK with any of these diseases (including tick transmission)?” Note that the widely varying response rate is due to the individual diseases only being visible to those participants who had responded that they had heard of that disease in the previous question. Correct answers are highlighted in bold.

Disease (*n* = Number of Respondents)	Yes, Infected Puppy Could Infect Another Dog *n* (%)	No, Infected Puppy Could Not Infect Another Dog *n* (%)	Not Sure *n* (%)	Don’t Know *n* (%)
Rabies (*n* = 5256)	**5061 (96.3%)**	8 (0.2%)	125 (2.4%)	62 (1.2%)
Brucellosis (Brucella; *n* = 3764)	**2913 (77.4%)**	17 (0.5%)	352 (9.4%)	138 (3.7%)
Leishmaniasis (Leishmania; *n* = 2843)	**2011 (70.7%)**	332 (11.7%)	411 (14.5%)	89 (3.1%)
Babesiosis (Babesia; *n* = 1771)	**1271 (71.8%)**	141 (8.0%)	309 (17.4%)	50 (2.8%)
Ehrlichiosis (Ehrlichia; *n* = 1383)	**918 (66.4%)**	138 (10.0%)	278 (20.1%)	49 (3.5%)
Hepatazoonosis (Hepatozoon; *n* = 1191)	**842 (70.7%)**	75 (6.3%)	238 (20.0%)	36 (3.0%)
Heartworm (Dirofiliaria; *n* = 4508)	2968 (65.8%)	**455 (10.0%)**	857 (19.0%)	228 (5.1%)

## Data Availability

The data presented in this study are available on request from the corresponding author.

## Supplementary Materials

The following supporting information can be downloaded at: https://www.mdpi.com/article/10.3390/ani15152193/s1, File S1: Final complete survey; Table S1: Univariable analysis of risk factor analysis for the number of questions about Pet Passports answered correctly. Variables with *p* < 0.2 are emboldened; Table S2: Univariable analysis of risk factor analysis for the number of diseases respondents had heard of variables with *p* < 0.2 are emboldened.
