# Supplementary material for: Knowledge of UK Residents About Importing Puppies from EU Countries
Source: Animals (Basel). 2025 Jul 25;15(15):2193. doi: 10.3390/ani15152193 (PMC12345551; doi:10.3390/ani15152193)
Supplement: Supplementary file 1 [file animals-15-02193-s001.zip › Table S1. Univariable analysis of risk factor analysis for pet passports questions.pdf]

	Variable (n=)	Category	n=	Coefficient	Standard error	t	95% CI	p
**Respondent demographic**	Current dog owner (n=7187)	Yes	5932	0.210	0.066	3.202	0.081 – 0.038	**0.001**
		**No**	1245	Ref				
	Previous dog owner (n=1282)	Yes	795	-0.130	0.122	-1.063	-0.371 - 0.110	0.288
		**No**	487	Ref				
	Considering new dog (n=7172)	Yes	1733	-0.081	0.057	-1.414	-0.193- 0.031	**0.157**
		**No**	5439	Ref				
	Age current dog acquired (n=6605)	>16 weeks	4801	0.236	0.057	4.134	0.124 - 0.348	**<0.001**
		<16 weeks	1804	Ref				
	Year current dog acquired (n=6650)	2022-2023	1841	0.237	0.104	2.282	0.033 – 0.441	**0.023**
		2020-2021	1994	0.211	0.103	2.045	0.009 - 0.413	**0.041**
		2015-2019	1605	0.334	0.106	3.154	0.126 - 0.541	**0.002**
		2008-2014	702	0.161	0.121	1.326	-0.077 - 0.398	**0.185**
		2007 and before	508	Ref				
	Owned non-UK born dog (n=4118)	Yes	588	0.914	0.089	10.321	0.741 - 1.088	**<0.001**
		No	3530	Ref				
	Travelled abroad with a dog (n=5328)	Yes	560	1.514	0.088	17.216	1.341 – 1.686	**<0.001**
		No	4768	Ref				
	Previously an EU resident (n=5316)	Yes	421	0.563	0.103	5.475	0.361 – 0.764	**<0.001**
		No	4895	Ref				
	Previous resident outside EU (n=5316)	Yes	379	0.127	0.108	1.172	-0.085 – 0.338	0.241
		No	4937	Ref				
	Respondent age (*n* =5302)	18-24 years	312	-0.025	0.129	-0.190	-0.278 – 0.229	0.849
		25-34 years	1130	0.397	0.086	4.625	0.229 - 0.565	**<0.01**
		35-44 years	1067	Ref				
		45-54 years	1059	-0.084	0.100	-0.794	-0.255 – 0.088	0.338

		55-64 years	1077	-0.059	0.087	-0.681	-0.230 – 0.111	0.496
		65+ years	657	-0.079	-0.794	0.100	-0.276 – 0.117	0.427
	Respondent gender (n=5249)	Female	4894	0.335	0.111	3.016	0.117 – 0.553	**0.003**
		Male	355	Ref				
	Worked with EU dogs (n=5321)	Yes	1132	1.926	0.062	30.920	1.804 – 2.048	**<0.01**
		No	4191	Ref				
	Current country of residency (n=5323)	England	3863	0.278	0.068	4.096	0.145 – 0.411	**<0.001**
		Scotland	1146	Ref				
		Wales	258	0.289	0.139	2.081	0.017 – 0.561	**0.038**
		Northern Ireland	53	0.537	0.285	1.886	-0.021 – 1.095	**0.059**
	Veterinary surgeon role (n=1123)	Yes	345	1.117	0.107	10.488	0.908– 1.326	**<0.001**
		No	778	Ref				
	Veterinary nurse role (n=1123)	Yes	324	0.160	0.113	1.411	-0.063 – 0.383	**0.158**
		No	799	Ref				
	Shelter worker role (n=1123)	Yes	149	-0.674	0.150	-4.482	-0.969- -0.380	**<0.001**
		No	974	Ref				
	Dog trainer role (n=1123)	Yes	125	-0.872	0.161	-5.416	-1.188 –0.056	**<0.001**
		No	998	Ref				

Table S1: Univariable analysis of risk factor analysis for the number of questions about pet passports answered correctly. Variables with p<0.2 are emboldened
