# Supplementary material for: Knowledge of UK Residents About Importing Puppies from EU Countries
Source: Animals (Basel). 2025 Jul 25;15(15):2193. doi: 10.3390/ani15152193 (PMC12345551; doi:10.3390/ani15152193)
Supplement: Supplementary file 1 [file animals-15-02193-s001.zip › Table S2. Univariable analysis of risk factors for the number of diseases respondents had heard of.pdf]

	Variable (n=)	Category	n=	Coefficient	Standard error	t	95% CI	p
**Respondent demographic**	Current dog owner (n=7187)	Yes	5932	-0.076	0.069	-1.094	-0.212– 0.060	0.274
		**No**	1245	Ref				
	Previous dog owner (n=1282)	Yes	795	-0.296	0.139	-2.124	-0.569 - -0.023	**0.034**
		**No**	487	Ref				
	Considering new dog (n=7172)	Yes	1733	0.073	0.060	1.205	-0.046 – 0.191	0.228
		**No**	5439	Ref				
	Age current dog acquired (n=6605)	>16 weeks	4801	0.353	0.060	5.867	0.235 – 0.471	**<0.01**
		<16 weeks	1804	Ref				
	Year current dog acquired (n=6650)	2022-2023	1841	0.172	0.109	1.575	-0.047 – 0.387	**0.115**
		2020-2021	1994	-0.063	0.108	-0.583	-0.274 – 0.148	0.560
		2015-2019	1605	0.360	0.111	3.238	0.142 – 0.577	**0.001**
		2008-2014	702	0.366	0.127	2.877	0.117 – 0.616	**0.004**
		2007 and before	508	Ref				
	Owned non-UK born dog (n=4118)	Yes	588	0.828	0.081	10.211	0.669 – 0.987	**<0.001**
		No	3530	Ref				
	Travelled abroad with a dog (n=5328)	Yes	560	0.658	0.085	7.776	0.492 – 0.824	**<0.01**
		No	4768	Ref				
	Previously an EU resident (n=5316)	Yes	421	0.327	0.097	3.381	0.137 – 0.516	**<0.001**
		No	4895	Ref				
	Previous resident outside EU (n=5316)	Yes	379	0.236	0.101	2.332	0.038 – 0.435	**0.020**
		No	4937	Ref				
	Respondent age (*n* =5302)	18-24 years	312	0.417	0.122	3.431	0.179 – 0.655	**<0.001**
		25-34 years	1130	0.550	0.081	6.819	0.392 – 0.708	**<0.001**

		35-44 years	1067	Ref				
		45-54 years	1059	-0.138	0.082	-1.690	-0.299 – 0.022	**0.091**
		55-64 years	1077	-0.097	0.082	-1.184	-0.257 – 0.063	0.237
		65+ years	657	0.060	0.094	0.640	-0.124 – 0.244	0.522
	Respondent gender (n=5249)	Female	4894	0.644	0.104	6.162	0.439 – 0.849	**<0.001**
		Male	355	Ref				
	Worked with EU dogs (n=5321)	Yes	1132	2.457	0.054	45.358	2.351 – 2.563	**<0.001**
		No	4191	Ref				
	Current country of residency (n=5323)	England	3863	0.472	0.056	7.411	0.347 – 0.597	**<0.001**
		Scotland	1146	Ref				
		Wales	258	0.482	0.131	3.689	0.226 – 0.738	**<0.001**
		Northern Ireland	53	-0.007	0.266	-0.028	-0.529 – 0.514	0.978
	Veterinary surgeon role (n=1123)	Yes	345	1.281	0.082	15.568	1.120 - 1.443	**<0.001**
		No	778	Ref				
	Veterinary nurse role (n=1123)	Yes	324	0.230	0.092	2.495	0.049 – 0.410	**0.013**
		No	799	Ref				
	Shelter worker role (n=1123)	Yes	149	-0.372	0.123	-3.029	-0.613 – -0.131	**0.003**
		No	974	Ref				
	Dog trainer role (n=1123)	Yes	125	-0.872	0.161	-5.416	-1.188 – 0.056	**<0.001**
		No	998	Ref				

Table S2. Univariable analysis of risk factor analysis for the number of diseases respondents had heard of. Variables with p<0.2 are emboldened
